# PSD3 as a context-dependent modulator of immune landscape and tumor aggressiveness in esophageal squamous cell carcinoma

**DOI:** 10.3389/fimmu.2025.1641254

**Published:** 2025-08-15

**Authors:** Shujuan Luo, Huifang Li, Bangwu Cai, Aididar Nurbahati, Hong Cui, Tianyuan Peng, Wei Wang, Qing Liu, Xiaomei Lu, Shutao Zheng

**Affiliations:** ^1^ State Key Laboratory of Pathogenesis, Prevention, Treatment of Central Asian High Incidence Diseases, Clinical Medical Research Institute, First Affiliated Hospital of Xinjiang Medical University, Urumqi, China; ^2^ Department of Breast Surgery, The First Affiliated Hospital of Xinjiang Medical University, Urumqi, Xinjiang, China; ^3^ Department of Digestive Internal Medicine, The Affiliated Tumor Hospital of Xinjiang Medical University, Urumqi, Xinjiang, China

**Keywords:** esophageal squamous cell carcinoma, PSD3, PD-L1, TNFSF18, tumor microenvironment, immune infiltration, biomarker, multiplex immunohistochemistry

## Abstract

In this study, we investigated PSD3, CD274 (PD-L1), and TNFSF18 as potential immune-related biomarkers in esophageal squamous cell carcinoma (ESCC) using integrative transcriptomic and experimental approaches. CD274 and TNFSF18 were consistently up-regulated in ESCC across both TCGA and GEO datasets, while PSD3 showed significantly higher expression in TCGA but no significant difference in the GEO cohort. Only PSD3 demonstrated a significant association with overall survival, with higher expression correlating with improved prognosis. Interestingly, despite its favorable prognostic value, PSD3 functionally promoted ESCC cell proliferation, invasion, and migration *in vitro*, while inversely regulating PD-L1 expression. Conversely, heterozygous knockout of PD-L1 in KYSE150 cells impaired tumor aggressiveness. Co-immunoprecipitation revealed a direct physical interaction between PSD3 and PD-L1, suggesting a regulatory axis with implications for immune evasion. These findings position PSD3 as a context-dependent immuno-oncogenic factor and a potential therapeutic target in ESCC.

## 1 Introduction

Esophageal cancer is among the most fatal malignancies worldwide, ranking seventh in incidence and sixth in cancer-related mortality globally (1). Esophageal squamous cell carcinoma (ESCC) represents the predominant histological subtype, particularly in East Asia, including China, where it accounts for over 90% of all esophageal cancer cases (2). Despite advances in endoscopic screening, surgical techniques, and chemoradiotherapy, the prognosis for ESCC remains dismal, with a five-year survival rate below 20% for most patients (3). This poor outcome is largely due to late diagnosis, high recurrence rates, and resistance to conventional treatments (4). Therefore, the identification of reliable molecular biomarkers for early detection, prognosis prediction, and therapeutic targeting is an urgent priority in the clinical management of ESCC.

In recent years, the rise of cancer immunotherapy has underscored the critical role of the tumor immune microenvironment in cancer progression and treatment response (5, 6). Notably, immune checkpoint inhibitors targeting PD-1/PD-L1 have demonstrated some efficacy in ESCC, yet only a subset of patients respond favorably (7, 8), highlighting the need for additional immune-related biomarkers to guide precision medicine. In this context, bioinformatic or *in silico* analyses of large-scale public datasets, such as those provided by The Cancer Genome Atlas (TCGA) (9), offer a powerful means to uncover novel candidate genes involved in ESCC pathogenesis and immunity. Such analyses allow the simultaneous evaluation of gene expression, clinical relevance, immune correlation, and pathway enrichment, facilitating the identification of genes with both prognostic and functional significance.

In our study, we focused on three genes—PSD3 (10), CD274 (also known as PD-L1) (11), and TNFSF18 [also known as GITRL (12)]—which are implicated in immune regulation and cancer biology. CD274 is a well-established immune checkpoint molecule that plays a pivotal role in immune escape mechanisms by inhibiting T cell activity in the tumor microenvironment. Its clinical relevance is underscored by its status as a therapeutic target in several cancers, including ESCC. TNFSF18, a member of the tumor necrosis factor superfamily, acts as a co-stimulatory molecule capable of enhancing T cell responses (13). Although less well-characterized, it has emerged as a potential immune modulator in the tumor milieu. PSD3 (Pleckstrin and Sec7 Domain Containing 3), by contrast, has received limited attention in cancer research. Preliminary studies suggest its involvement in intracellular signaling and cytoskeletal reorganization (14), and its expression has been observed in several cancers (10, 15), yet its precise role in ESCC and tumor immunity remains largely unexplored.

Despite the clinical and biological relevance of these genes, their integrated expression patterns, prognostic impact, immune associations, and mechanistic pathways in ESCC have not been systematically examined. This knowledge gap limits the effective stratification of patients for immunotherapy and the development of novel therapeutic strategies. To address this, we employed comprehensive *in silico* approaches to investigate the expression, prognostic value, functional enrichment, and immune correlations of PSD3, CD274, and TNFSF18 in ESCC using TCGA datasets. In addition to computational analyses, we performed multiplex immunohistochemistry and functional assays to validate key findings at the protein and phenotypic levels. This integrated approach enables a more comprehensive understanding of PSD3, CD274, and TNFSF18 in the context of the ESCC tumor immune microenvironment.

Through this study, we aim to determine whether PSD3, CD274, and TNFSF18—individually or as a panel—could serve as effective biomarkers for ESCC prognosis and immune status. Our findings not only expand the understanding of immune-related gene expression in ESCC but also provide a foundation for future studies on immunotherapeutic targets and precision oncology in esophageal cancer.

In this study, we selected PSD3, CD274, and TNFSF18 for integrated analysis based on a combination of transcriptomic screening, immunological relevance, and prognostic associations in esophageal squamous cell carcinoma (ESCC). PSD3 emerged from our pan-cancer expression and co-expression analyses as a novel and previously uncharacterized gene in ESCC, showing strong associations with immune-related pathways and clinical outcomes. CD274 (also known as PD-L1), a well-established immune checkpoint molecule, was included as a benchmark and functional comparator to contextualize PSD3’s potential role in immune evasion. TNFSF18, a member of the tumor necrosis factor superfamily known to modulate T cell activity, was selected based on its emerging links to tumor immunity and its co-regulation patterns with PSD3. Together, these three genes span a spectrum from novel (PSD3) to canonical (CD274), allowing us to explore shared and distinct immunological and oncogenic mechanisms in ESCC.

## 2 Materials and methods

### 2.1 Data acquisition and preprocessing

Transcriptomic expression data and corresponding clinical information for esophageal squamous cell carcinoma (ESCC) were obtained from The Cancer Genome Atlas (TCGA) via the UCSC Xena platform (https://xenabrowser.net/) as well as from the Gene Expression Omnibus (GEO) under accession number GSE23400. For the TCGA ESCC cohort, a total of 95 ESCC samples were included. In addition, the normal esophageal mucosa tissues, totaling 269, retrieved from Genotype-Tissue Expression (GTEx) (16) database. The GEO dataset GSE23400, generated using the Affymetrix Human Genome U133 plus 2.0 array platform, comprised 53 paired tumor and adjacent normal samples from Chinese ESCC patients. Gene expression data from TCGA were normalized to transcripts per million (TPM) format (17), while the GEO microarray data were preprocessed using robust multi-array average (RMA) normalization (18). Only samples with complete clinical annotation and valid expression profiles for PSD3, CD274, and TNFSF18 were retained for downstream analyses.

### 2.2 Gene expression and survival analyses

Expression patterns of PSD3, CD274, and TNFSF18 were compared between tumor and normal tissues using non-parametric Mann–Whitney U tests due to non-normal data distribution. Statistical significance was set at p < 0.05. Kaplan–Meier survival curves were generated to assess the relationship between gene expression and overall survival (OS) in ESCC patients. Patients were dichotomized into high- and low-expression groups based on the median expression level of each gene. Log-rank tests were applied to evaluate differences in survival outcomes. All survival analyses were conducted using the R package survival (version 3.5-5) (19).

### 2.3 Association with clinical features and diagnostic value

To explore associations between gene expression and clinicopathological variables, including tumor (T), node (N), metastasis (M) stages and clinical staging, Mann–Whitney U tests were employed. Receiver operating characteristic (ROC) curve analyses were performed using the pROC package (20) in R to assess the diagnostic performance of each gene in distinguishing tumor from normal tissues. The area under the curve (AUC), sensitivity, and specificity were calculated to evaluate diagnostic efficacy.

### 2.4 Co-expression and functional enrichment analysis

Pearson correlation analysis was conducted to identify the top genes most positively associated with PSD3, CD274, and TNFSF18 in ESCC samples. The top 200 co-expressed genes for each target were subjected to Gene Ontology (GO) enrichment analysis using the clusterProfiler package (21) (version 4.6.2). Enrichment was performed across biological processes, cellular components, and molecular functions with a significance threshold of adjusted p < 0.05 using the Benjamini-Hochberg method. Additionally, Gene Set Enrichment Analysis (GSEA) was carried out to explore Reactome pathway involvement, using the gene set enrichment (GSE) pathway function (22). Z-score normalization (23) was applied prior to visualization of gene expression patterns.

### 2.5 Immune infiltration correlation analysis

To examine the relationship between gene expression and immune cell infiltration, we utilized the ImmuCellAI (24) and TIMER2.0 (25) databases for immune deconvolution data. Correlation coefficients were calculated between gene expression levels and the relative abundance of 22 immune cell types, including CD8+ T cells, macrophages, and dendritic cells. Heatmaps were generated to visualize the strength and direction of these associations. Stratified analyses were also performed to compare immune cell infiltration in high- and low-expression groups for each gene using the Mann–Whitney U test.

### 2.6 AKR and KYSE150 cell culture and lentiviral transfection

Murine ESCC AKR cells and human ESCC KYSE150 cells were cultured in DMEM or RPMI 1640 medium (Gibco, Thermo Fisher Scientific, USA), respectively, each supplemented with 10% fetal bovine serum (FBS; Gibco), 1% penicillin-streptomycin (Gibco), and maintained in a humidified incubator at 37°C with 5% CO_2_. Lentiviral vectors encoding PSD3-targeting short hairpin RNAs (shRNAs) or scramble control (control) were commercially purchased from GeneChem (Shanghai, China). Heterozygous knockout of PD-L1 in KYSE150 cells was performed by Ubigene (Guangzhou, China) as a scientific service. Transfections were carried out at a multiplicity of infection (MOI) of 20 in the presence of 8 μg/mL polybrene. AKR cells were incubated with viral particles for 24 hours, followed by replacement with fresh medium. Puromycin (2 μg/mL) was added 72 hours post-infection to select for stably transduced cells.

### 2.7 RNA extraction and quantitative real-time PCR

Total RNA was isolated from cells using the RNA Extraction Kit provided by Foregene (Chengdu, China; Cat. No. RE-03111). The extracted RNA was immediately reverse transcribed using the PrimeScript RT Master Mix (Cat. No. RR036A). Subsequent amplification of specific RNA targets was carried out using the SYBR Premix Ex Taq II kit (TaKaRa). For the mouse PSD3 gene, the forward primer was ACCCTCAAGTCCCAGCCTCAAG, and the reverse primer was CTCTCCACTGCCCTAGCCTCAC; for human PD-L1, the forward primer was GTGGGATGCAGGCAATGTGG, the reverse primer was TCAAGGTCTCCCTCCAGGCT. For human 18S rRNA, the forward primer was ATCCTCAGTGAGTTCTCCCG, the reverse primer was CTTTGCCATCACTGCCATTA. For murine β-actin, the forward and reverse primers were GTGCTATGTTGCTCTAGACTTCG and ATGCCACAGGATTCCATACC, respectively. The PCR protocol began with an initial denaturation at 95°C for 5 minutes, followed by 40 cycles consisting of 5 seconds at 95°C and 34 seconds at 58°C. Relative expression levels were quantified using the 2^–ΔΔCT^ method, with murine β-actin and human 18S rRNA serving as the internal control.

### 2.8 Western blot

Briefly, proteins (30 μg per lane) were separated by 10% SDS-PAGE and then transferred onto PVDF membranes. Afterward, membranes were blocked for 1.5 hours at room temperature using 5% BSA in TBST. Primary antibodies, including anti-mouse β-actin (Catalog No. 66009-1-Ig, 1:1000; Proteintech, Wuhan, China) and anti-mouse PSD3 (1:1000; BYab-09310, Boyan Biology, Nanjing, China), PD-L1 (1:1000; 66248-1-lg, Proteintech) were diluted in TBST and incubated with the membranes overnight at 4°C. The following day, membranes were treated with HRP-conjugated secondary antibodies specific to mouse or rabbit IgG (bs-0295G-AP; Bioss, China) for 1.5 hours at ambient temperature. Protein signals were detected using enhanced chemiluminescence (ECL) reagents and visualized using the eBLOT Touch Imager (eBLOT, Shanghai, China).

### 2.9 Clonogenic assay

To assess clonogenic capacity, AKR cells (control and shRNA-PSD3 knockdown) were seeded at a density of 500 cells per well in 6-well plates and cultured for 14 days. At the endpoint, colonies were fixed with 4% paraformaldehyde for 15 minutes, stained with 0.5% crystal violet (Sigma-Aldrich) for 30 minutes, and gently rinsed with distilled water. Colonies consisting of >50 cells were counted manually under a light microscope. Each experiment was performed in triplicate.

### 2.10 EdU proliferation assay

Cell proliferation was measured using the Cell-Light™ EdU Apollo567 *In Vitro* Imaging Kit (RiboBio, Guangzhou, China) according to the manufacturer’s protocol. Briefly, 2 × 10^4^ AKR cells were seeded onto sterile coverslips in 24-well plates and cultured for 24 hours, followed by incubation with 50 μM EdU for 2 hours. Cells were then fixed with 4% paraformaldehyde, permeabilized with 0.5% Triton X-100, and stained with Apollo567 and DAPI. Fluorescence images were captured using a confocal fluorescence microscope (Leica, Germany), and EdU-positive cells were quantified as a percentage of total DAPI-stained nuclei.

### 2.11 Wound-healing assay

AKR cells were seeded in 6-well plates and grown to ~90% confluence. A sterile 200-μL pipette tip was used to create a linear scratch across the monolayer. After washing with PBS to remove detached cells, fresh serum-free DMEM was added. Images of the same wound area were captured at 0 and 24 hours using an inverted microscope (Olympus, Tokyo, Japan), and migration rate was calculated using ImageJ software.

### 2.12 Transwell invasion assay

Transwell chambers with 8-μm pore membranes (Corning Costar, USA) were coated with Matrigel (BD Biosciences) diluted 1:8 in serum-free medium. A total of 1 × 10^5^ cells in 200 μL serum-free medium were seeded into the upper chambers, while 600 μL of medium containing 10% FBS was added to the lower chambers as a chemoattractant. After 24 hours of incubation, cells that had invaded through the Matrigel were fixed with 4% paraformaldehyde, stained with 0.5% crystal violet, and counted in five random fields per insert under an inverted microscope (Olympus, Tokyo, Japan).

### 2.13 Multiplex immunohistochemistry

ESCC tissue microarray slides were first dewaxed and rehydrated, followed by antigen retrieval. Slides were then blocked with 2% goat serum and stained using the TG TSA Multiplex IHC Assay Kit (TissueGnostics Asia-Pacific Ltd.). Primary antibodies included pan-cytokeratin (pan-CK, ab7753, Abcam; 1:4000 dilution), PSD3 (BYab-09310, Boyan Biology, Nanjing, China; 1:1000 dilution), and PD-L1 (66248-1-Ig; Proteintech, Wuhan, China). A secondary antibody from Zhongshan Goldbridge Biotechnology (China; pre-diluted) and the NEON E-TSA Smart540 seven-color kit (HISTOVA, Beijing Histova Biotechnology Co., Ltd.) were applied subsequently. Nuclear counterstaining was performed with DAPI. Quantitative analysis of cell density, nuclear area per cell, and marker expression per cell was conducted using StrataQuest software (v7.1.119; TissueGnostics GmbH, Vienna, Austria).

### 2.14 Statistical analyses

All statistical analyses were conducted using R (version 4.2.2) and SPSS (version 26.0; IBM Corp., Armonk, NY, USA). Data are presented as mean ± standard deviation (SD). The Mann–Whitney U test was consistently used to compare two independent groups due to the non-parametric nature of the datasets. A two-tailed p value < 0.05 was considered statistically significant unless otherwise stated. Figures and plots were generated using ggplot2, pheatmap, and ComplexHeatmap packages in R.

## 3 Results

### 3.1 Differential expression of PSD3, CD274, and TNFSF18 in pan-cancer and ESCC

**Figure 1:**
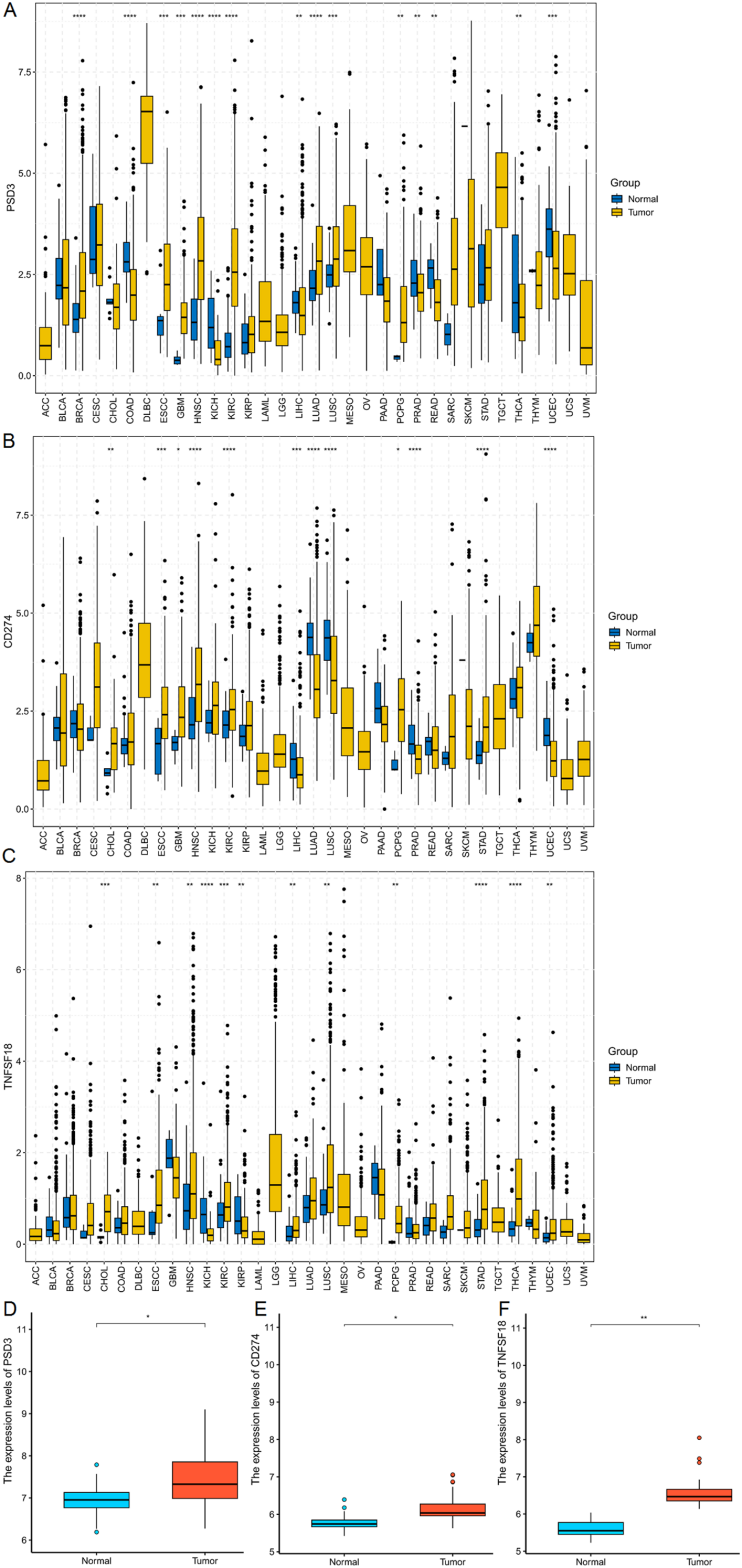
mRNA expression analysis of PSD3, CD274, and TNFSF18 in Pan-cancer and ESCC. **(A–C)** Comparative analysis of PSD3, CD274, and TNFSF18 mRNA expression levels in tumor versus normal tissues across various cancer types, based on data from The Cancer Genome Atlas (TCGA). **(D–F)** Expression profiles of PSD3, CD274, and TNFSF18 specifically in esophageal squamous cell carcinoma (ESCC), also derived from TCGA datasets. Data are presented as mean ± standard deviation (SD). Statistical significance was determined using the Mann–Whitney U test due to the non-normal distribution of the data. *P < 0.05, **P < 0.01, ***P < 0.001, ****P<0.0001.

To establish a foundational understanding of PSD3, CD274 (PD-L1), and TNFSF18 expression in cancer, we first conducted a comparative analysis of their mRNA levels across a wide array of tumor types using The Cancer Genome Atlas (TCGA) datasets. All three genes exhibited variable expression patterns in tumor tissues versus normal counterparts across multiple malignancies, suggesting their potential involvement in diverse oncogenic processes (**Figures 1A–C**). When specifically examined within esophageal squamous cell carcinoma (ESCC), each gene displayed significantly elevated expression in tumor tissues compared to adjacent normal tissues (P < 0.001 for all; **Figures 1D–F**). These consistent overexpression patterns in ESCC laid the groundwork for subsequent investigations into their clinical relevance, prognostic value, and immunological function.

### 3.2 Prognostic implications and clinical associations in ESCC based on TCGA data

**Figure 2:**
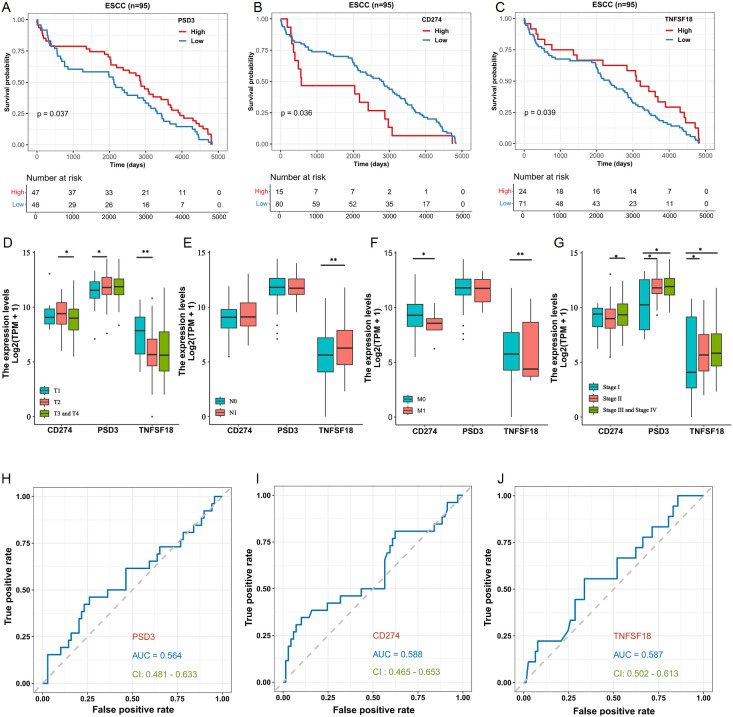
Prognostic and clinicopathological Analyses of PSD3, CD274, and TNFSF18 expression in relation to overall survival (OS) in ESCC based on TCGA data. **(A–C)** Kaplan–Meier survival analyses illustrating the association between the expression levels of PSD3, CD274, and TNFSF18 and overall survival in esophageal squamous cell carcinoma (ESCC), based on TCGA data. **(D–G)** Correlation analyses between PSD3, CD274, and TNFSF18 expression levels and clinicopathological parameters, including T (tumor size), N (lymph node involvement), M (metastasis), and overall clinical stage in ESCC. **(H–J)** Diagnostic performance of PSD3, CD274, and TNFSF18 assessed by receiver operating characteristic (ROC) curve analysis in ESCC. Data are presented as mean ± standard deviation (SD). Statistical comparisons were performed using the Mann–Whitney U test due to non-normal data distribution. *P < 0.05, **P < 0.01.

Building on the observed over-expression in ESCC, we next explored whether PSD3, CD274, and TNFSF18 might serve as prognostic biomarkers. Kaplan–Meier survival analysis using the TCGA-ESCC cohort demonstrated that elevated expression levels of CD274 and TNFSF18 were significantly correlated with shorter overall survival (log-rank P < 0.05; **Figures 2B, C**). Interestingly, and contrary to expectation, higher expression of PSD3 was significantly associated with longer overall survival (log-rank P < 0.05; **Figure 2A**). These findings suggest that CD274 and TNFSF18 may serve as unfavorable prognostic markers, whereas PSD3 may indicate a more favorable prognosis. We further investigated the correlation between gene expression and clinical staging parameters, including tumor size (T), nodal involvement (N), distant metastasis (M), and overall clinical stage. PSD3 exhibited a significantly progressive up-regulation in T1 and T2 stages, but this trend was not observed in T3 and T4 (**Figure 2D**). In contrast, CD274 showed a slight up-regulation in T1 and T2, followed by a significant down-regulation in T3 and T4. As for TNFSF18, its mRNA expression was markedly reduced in T1 and T2, reaching a plateau in the later stages (T3 and T4) (**Figure 2D**). With regard to N classification, no significant association was found for CD274 and PSD3 expression. However, TNFSF18 showed a striking increase in expression in N0 and N1 stages (**Figure 2E**). In terms of M classification, both CD274 and TNFSF18 demonstrated notable down-regulation in M0 and M1 groups. In contrast, PSD3 did not exhibit any significant association with M classification (**Figure 2F**). Regarding clinical stage, both PSD3 and TNFSF18 showed a progressively increasing expression from stage I through stage II to stages III–IV, indicating a significant upward trend. Conversely, CD274 expression was only significantly different between stage II and stages III–IV, without consistent trends across all stages (**Figure 2G**). Despite these fluctuations, all three genes demonstrated stage-dependent expression variability. ROC curve analysis supported their diagnostic utility, with AUCs of 0.564, 0.588, and 0.587 for PSD3, CD274, and TNFSF18, respectively (**Figures 2H–J**). These data collectively suggest PSD3 may serve as a more stable marker across clinical settings, while CD274 and TNFSF18 may reflect dynamic tumor-immune interactions.

### 3.3 External validation in GEO dataset confirms prognostic and diagnostic relevance

**Figure 3:**
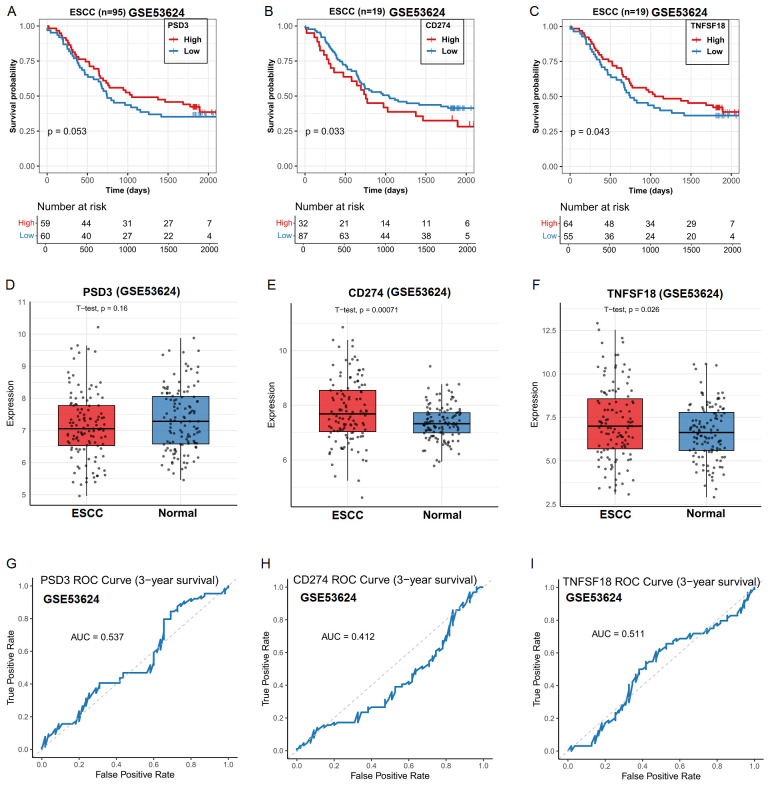
Prognostic and clinicopathological analysis of PSD3, CD274, and TNFSF18 expression in relation to overall survival (OS) in ESCC based on GEO data. **(A–C)** Kaplan–Meier survival curves showing the association between PSD3, CD274, and TNFSF18 expression levels and overall survival in ESCC, derived from the GEO dataset GSE53624. **(D–F)** Differential expression of PSD3, CD274, and TNFSF18 between ESCC tumor tissues and adjacent normal controls, based on GSE53624. **(G–I)** Diagnostic performance of PSD3, CD274, and TNFSF18 in distinguishing ESCC tumors from normal tissues, evaluated using receiver operating characteristic (ROC) curve analysis. Data are presented as mean ± standard deviation (SD). Group comparisons were conducted using the Mann–Whitney U test due to non-normal data distribution.

To validate the robustness of our TCGA-derived observations, we further analyzed the GSE53624 dataset from the Gene Expression Omnibus (GEO). In contrast to the TCGA findings, high PSD3 expression was significantly associated with improved overall survival in ESCC patients (P < 0.05; **Figure 3A**), indicating a potential protective role. In line with TCGA results, elevated expression levels of CD274 and TNFSF18 remained significantly correlated with poorer overall survival (P < 0.05; **Figures 3B, C**). However, differential expression analysis revealed divergent patterns. While CD274 and TNFSF18 were significantly over-expressed in ESCC tumor tissues compared to adjacent normal controls (P < 0.001; **Figure 3E, F**), PSD3 did not show a statistically significant difference between tumor and normal tissues in this cohort (**Figure 3D**). Receiver operating characteristic (ROC) curve analysis in this independent dataset further reflected these discrepancies. The diagnostic performance of PSD3, CD274, and TNFSF18 yielded modest AUC values of 0.537, 0.412, and 0.511, respectively (**Figures 3G–I**), suggesting limited diagnostic value for distinguishing ESCC from normal tissue in this cohort. Taken together, these findings highlight the cohort-dependent nature of these biomarkers and suggest that while CD274 and TNFSF18 may serve as consistent prognostic indicators, the prognostic and diagnostic implications of PSD3 warrant further investigation.

### 3.4 Gene expression correlates with Immune cell infiltration in ESCC

**Figure 4:**
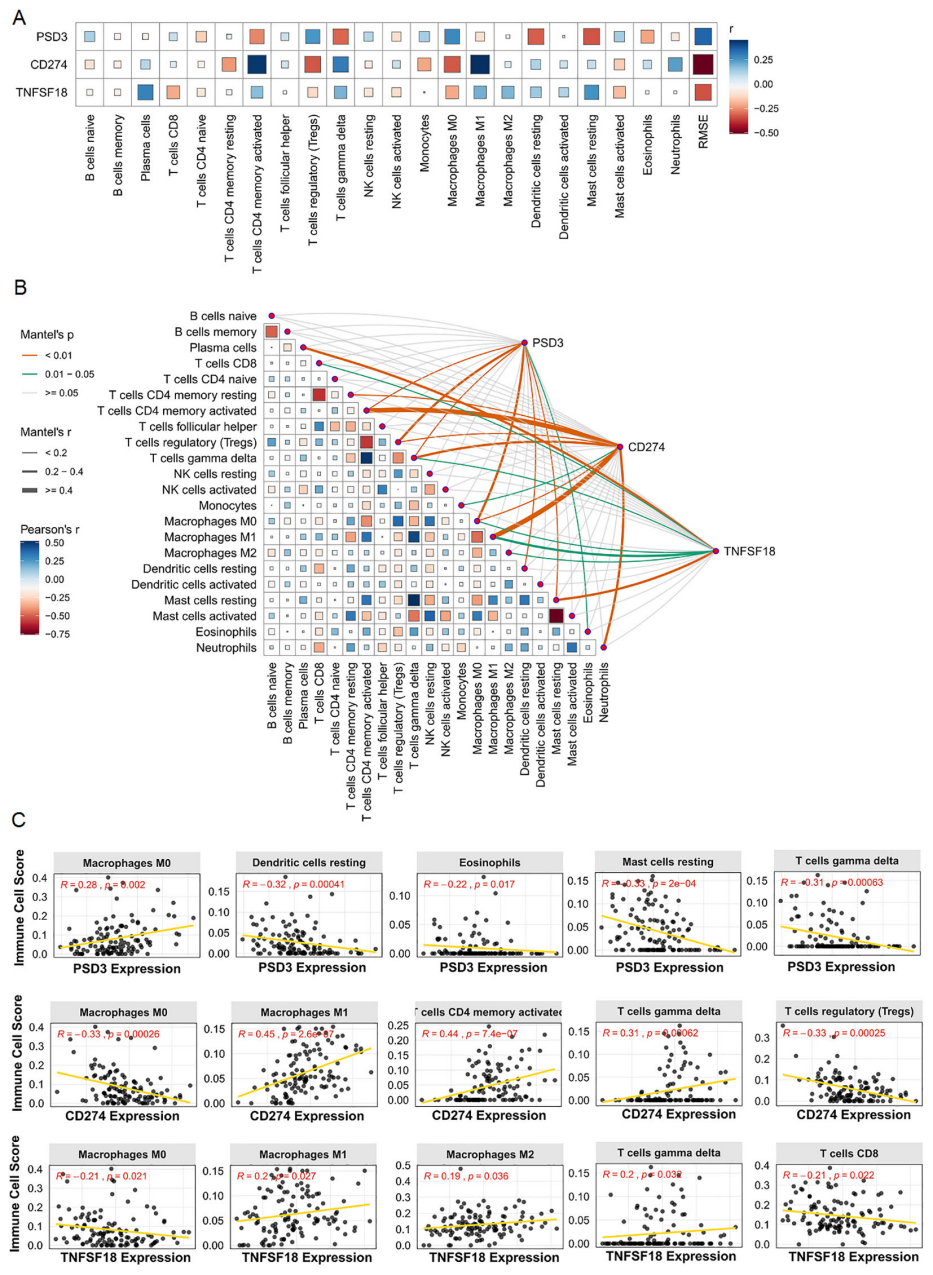
Correlation analysis between PSD3, CD274, and TNFSF18 expression and immune cell infiltration in ESCC based on GEO data. **(A)** Bar chart illustrating the correlation coefficients between the expression of PSD3, CD274, and TNFSF18 and various immune cell populations in ESCC. **(B)** Heatmap visualizing the strength and direction of correlations between gene expression and immune cell infiltration levels. **(C)** Scatter plots showing representative correlation analyses between PSD3, CD274, and TNFSF18 expression and specific immune cell types. Correlation coefficients were calculated using Pearson correlation to assess the relationship between gene expression and immune infiltration.

Given the immunomodulatory roles of CD274 and TNFSF18, along with the emerging relevance of PSD3 in cancer biology, we next investigated their associations with tumor-infiltrating immune cells using immune deconvolution data from the GEO dataset GEO dataset (**Figure 4A**). The correlation analyses revealed distinct immune landscapes linked to each gene. PSD3 expression showed a predominantly negative association with several immune cell types (**Figure 4B**). Specifically, it was inversely correlated with resting dendritic cells (R = –0.32, p = 0.00041), eosinophils (R = –0.22, p = 0.017), resting mast cells (R = –0.33, p = 2e-04), activated CD4 memory T cells (R = –0.26, p = 0.0038), and γδ T cells (R = –0.31, p = 0.00063). Interestingly, PSD3 was positively associated with M0 macrophages (R = 0.28, p = 0.002) and regulatory T cells (Tregs) (R = 0.25, p = 0.0056), suggesting a potential role in fostering an immune-suppressive microenvironment. In contrast, CD274 displayed an immune profile indicative of active immunomodulation. It was significantly positively correlated with pro-inflammatory immune cells, including M1 macrophages (R = 0.45, p = 2.6e-07), neutrophils (R = 0.24, p = 0.0095), activated CD4 memory T cells (R = 0.44, p = 7.4e-07), and γδ T cells (R = 0.31, p = 0.00062). Conversely, it showed negative correlations with M0 macrophages (R = –0.33, p = 0.00026), monocytes (R = –0.22, p = 0.017), and notably, Tregs (R = –0.33, p = 0.00025). These findings point to a complex, potentially dual role for CD274 in modulating immune activation versus suppression. TNFSF18 presented a more modest pattern of associations. It was inversely correlated with M0 macrophages (R = –0.21, p = 0.021) and CD8+ T cells (R = –0.21, p = 0.022), both of which are often linked to effective antitumor responses. Meanwhile, positive correlations were observed with M1 macrophages (R = 0.20, p = 0.027), plasma cells (R = 0.30, p = 0.0011), and γδ T cells (R = 0.20, p = 0.032), indicating a nuanced involvement in shaping both adaptive and innate immune responses (**Figure 4C**). Together, these data underscore that PSD3, CD274, and TNFSF18 each associate with distinct immune cell infiltration profiles in ESCC. PSD3 appears to skew the tumor milieu toward immune suppression, while CD274 is linked to both stimulatory and suppressive immune components. TNFSF18 occupies a middle ground, exhibiting moderate correlations with both immune-activating and immune-dampening cell types. These gene-specific immune interactions may have critical implications for understanding ESCC immunobiology and informing therapeutic strategies. 

### 3.5 Co-expression networks and functional enrichment of correlated genes

**Figure 5:**
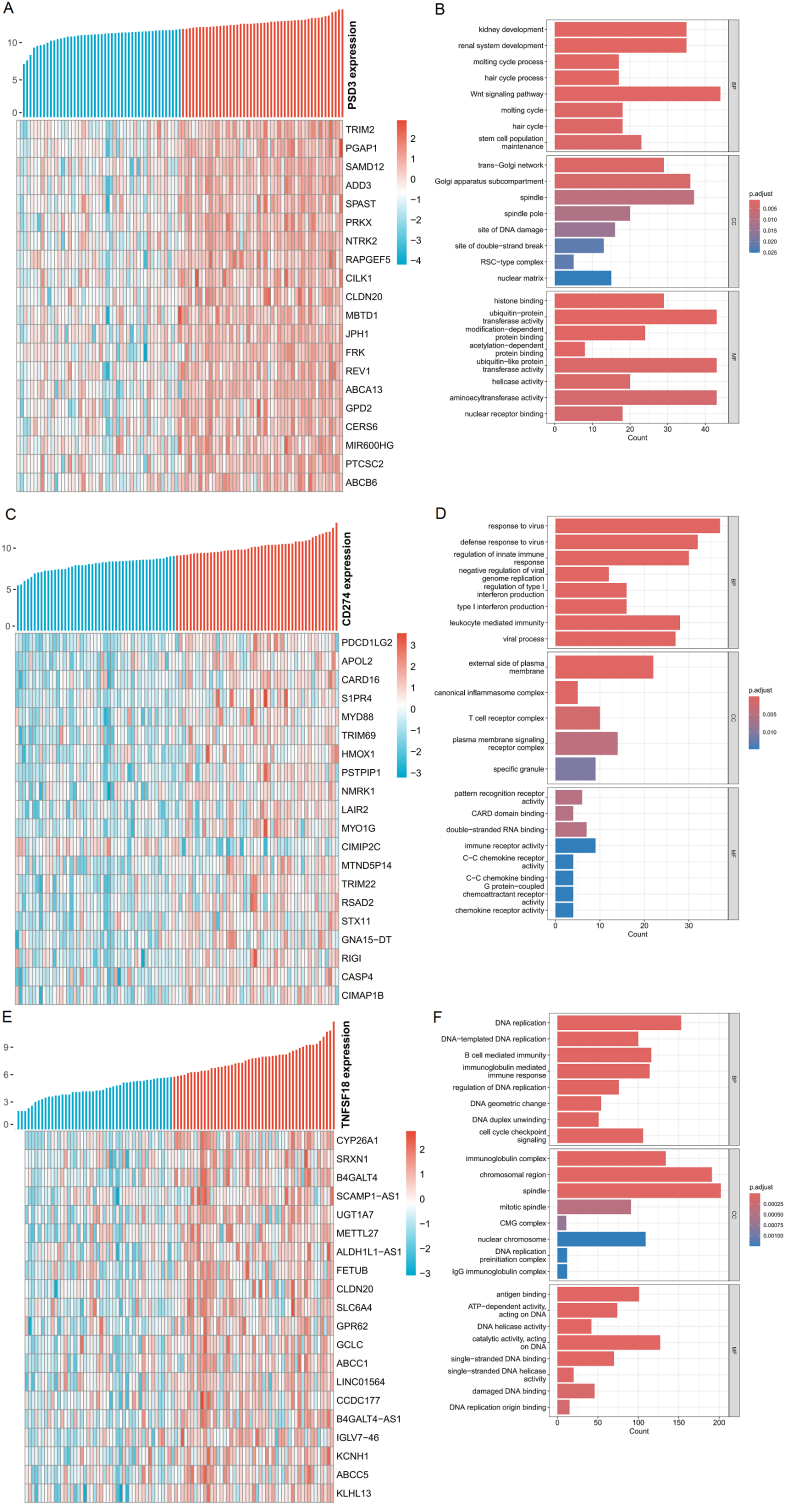
Analysis of genes positively associated with PSD3, CD274, and TNFSF18 expression in ESCC. **(A, C, E)** Heatmaps displaying the top 20 genes most positively correlated with PSD3, CD274, and TNFSF18 expression, respectively, in esophageal squamous cell carcinoma (ESCC). Gene expression values were normalized using Z-score standardization. **(B, D, F)** Gene Ontology (GO) enrichment analysis of the top 200 genes most positively correlated with PSD3, CD274, and TNFSF18 expression in ESCC, highlighting the biological processes potentially associated with their expression profiles.

To infer the potential biological roles of PSD3, CD274, and TNFSF18, we identified the top 200 genes most positively correlated with each in ESCC and performed Gene Ontology (GO) enrichment analysis. Heatmaps revealed coherent co-expression modules, with PSD3-associated genes enriched in cell cycle regulation, mitotic spindle organization, and cytoskeletal remodeling (**Figures 5A, B**). CD274-correlated genes were predominantly involved in interferon signaling, leukocyte activation, and antigen presentation pathways (**Figures 5C, D**), while TNFSF18 showed enrichment in T cell receptor signaling and NF-κB pathways (**Figures 5E, F**). These distinct enrichment profiles underscore the divergent functional landscapes of these genes: PSD3 appears to drive tumor-intrinsic processes, while CD274 and TNFSF18 modulate tumor-immune interactions.

### 3.6 Pathway-level insights via GSEA and immune signature heatmaps

**Figure 6:**
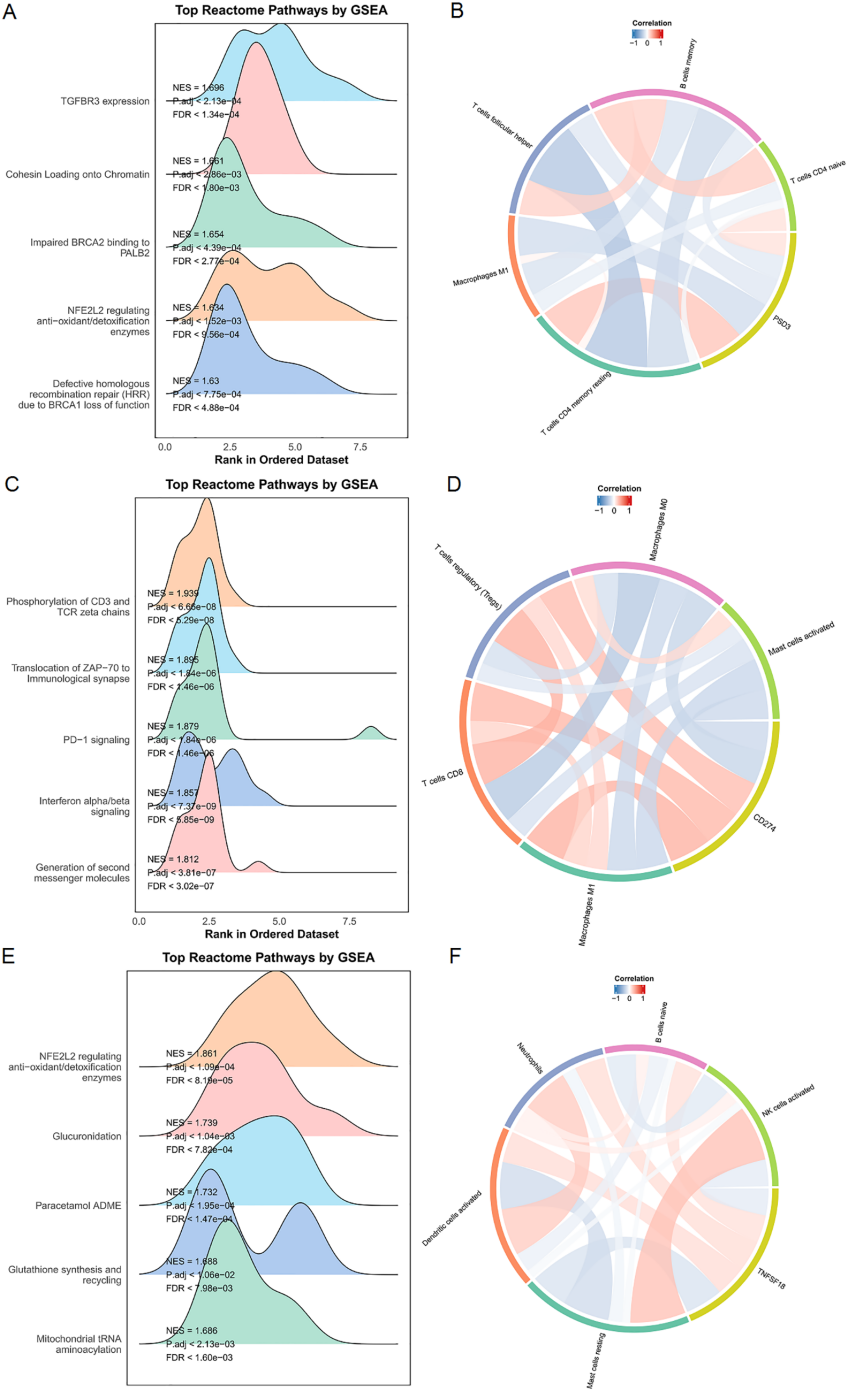
Enrichment and immune infiltration analyses of PSD3, CD274, and TNFSF18 in ESCC. **(A, C, E)** Gene Set Enrichment Analysis (GSEA) results for PSD3, CD274, and TNFSF18 in esophageal squamous cell carcinoma (ESCC), including enrichment in Reactome pathways, highlighting their potential functional roles. **(B, D, F)** Correlation analyses between immune cell infiltration levels and the expression of PSD3, CD274, and TNFSF18. Red indicates a positive correlation, while blue indicates a negative correlation; deeper color intensity corresponds to a stronger correlation. Data are presented as mean ± standard deviation (SD).

To complement the Gene Ontology (GO) analysis, we performed Gene Set Enrichment Analysis (GSEA) to explore pathway-level distinctions associated with high expression of PSD3, CD274, and TNFSF18. The results revealed that each gene was enriched in a unique set of biological processes, reflecting their potentially divergent roles in ESCC pathophysiology. In tumors with high PSD3 expression, enrichment was predominantly observed in pathways related to genomic integrity and stress responses. Notably, top-ranked pathways included TGFBR3 expression (NES = 1.696, p < 2.1×10^-4^, FDR < 1.34×10^-4^), cohesin loading onto chromatin (NES = 1.661, p < 2.86×10^-3^, FDR < 1.80×10^-3^), and impaired BRCA2 binding to PALB2 (NES = 1.654, p < 4.39×10^-4^, FDR < 2.77×10^-4^) (**Figure 6A**). Additional enrichment in NFE2L2-regulated antioxidant/detoxification enzymes and defective homologous recombination repair (HRR) due to BRCA1 loss of function suggests that PSD3 may be intricately involved in DNA repair deficits and redox regulation, rather than classic immune-related signaling (**Figure 6B**). In contrast, the pathway landscape for CD274—a well-known immune checkpoint molecule—was heavily skewed toward immunological signaling cascades. The top-enriched pathways included phosphorylation of CD3 and TCR zeta chains (NES = 1.939, p < 6.66×10^-8^, FDR < 5.29×10^-8^), translocation of ZAP-70 to the immunological synapse (NES = 1.895, p < 1.84×10^-6^, FDR < 1.46×10^-6^), and PD-1 signaling (NES = 1.879, p < 1.84×10^-6^, FDR < 1.46×10^-6^) (**Figure 6C**). Also enriched were interferon alpha/beta signaling and generation of second messenger molecules, further underscoring CD274’s role in T cell activation and immune checkpoint dynamics within the tumor microenvironment (**Figure 6D**). Meanwhile, TNFSF18 exhibited a distinct enrichment profile focused on metabolic and detoxification-related processes. Top pathways included NFE2L2-regulated antioxidant/detoxification enzymes (NES = 1.861, p < 1.09×10^-4^, FDR < 8.19×10^-5^), glucuronidation (NES = 1.739, p < 1.04×10^-3^, FDR < 7.82×10^-4^), and paracetamol ADME (NES = 1.732, p < 1.95×10^-4^, FDR < 1.47×10^-4^) (**Figure 6E**). Additional enrichment in glutathione synthesis and recycling and mitochondrial tRNA aminoacylation points toward a possible role for TNFSF18 in redox balance and mitochondrial function (**Figure 6F**). In summary, while CD274 was tightly associated with classical immune signaling pathways, and TNFSF18 aligned with metabolic detoxification programs, PSD3 stood apart by linking primarily to DNA repair dysfunction and chromatin regulation. These distinct pathway associations highlight the non-overlapping biological roles of these genes and suggest diverse mechanisms through which they may influence ESCC progression and immune interactions.

### 3.7 Immune infiltration differences in high vs. low expression groups

**Figure 7:**
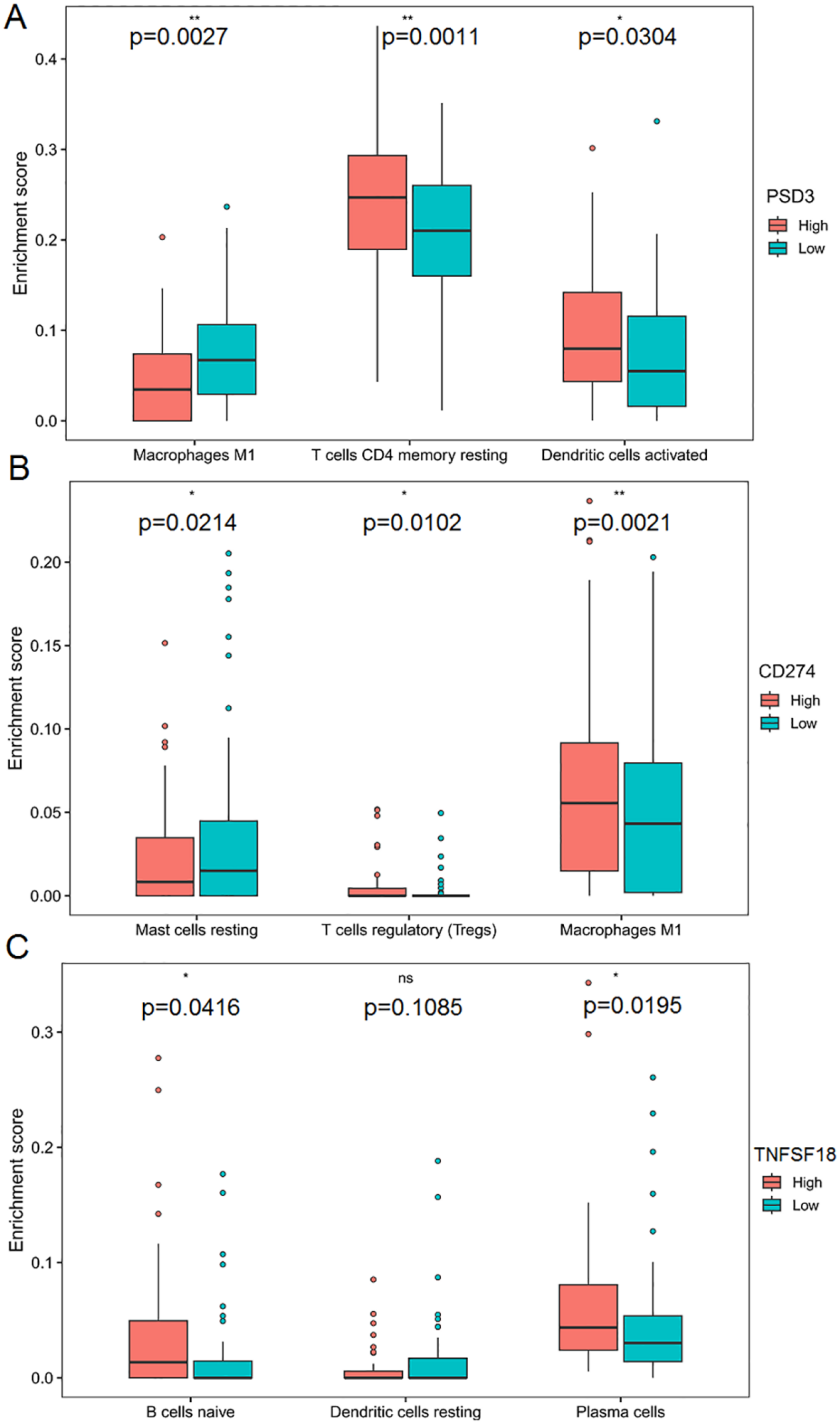
Correlation between PSD3, CD274, and TNFSF18 expression and immune cell infiltration in ESCC based on TCGA data. **(A)** Comparison of immune cell infiltration levels between high and low PSD3 expression groups in esophageal squamous cell carcinoma (ESCC), based on TCGA data. **(B, C)** Similar comparisons of immune cell infiltration between high and low expression groups for CD274 and TNFSF18, respectively, in ESCC. Statistical analysis was performed using the Mann–Whitney U test. *P < 0.05, **P < 0.01. ns, non-significant.

Stratifying TCGA-ESCC patients by gene expression levels revealed distinct immune cell infiltration patterns, highlighting gene-specific influences on the tumor immune microenvironment. High PSD3 expression was significantly associated with increased infiltration of M1 macrophages (P = 0.0027), resting CD4 memory T cells (P = 0.0011), and activated dendritic cells (P = 0.0304) (**Figure 7A**). This profile suggests a potential link between PSD3 and pro-inflammatory immune engagement, possibly contradicting earlier assumptions of an immune-desert phenotype. In contrast, elevated CD274 expression correlated with greater infiltration of resting mast cells (P = 0.0214), regulatory T cells (Tregs) (P = 0.0102), and M1 macrophages (P = 0.0021). While the presence of M1 macrophages may reflect some degree of immune activation, the simultaneous enrichment of Tregs and mast cells—both often associated with immunosuppressive environments—reinforces CD274’s complex, dual-role in immune modulation (**Figure 7B**). TNFSF18-high tumors demonstrated a different signature altogether. Significant associations were observed with naïve B cells (P = 0.0416) and plasma cells (P = 0.0195), suggesting a more prominent role in humoral immunity. Notably, TNFSF18 showed no significant correlation with resting dendritic cells (P = 0.1085), indicating limited involvement in antigen-presenting cell dynamics (**Figure 7C**). Together, these stratified analyses underscore the divergent immunological landscapes shaped by PSD3, CD274, and TNFSF18. While PSD3 aligns more closely with innate and memory T cell components, CD274 intersects with both activation and suppression arms of immunity, and TNFSF18 appears to influence B cell-mediated responses. These distinctions may hold clinical relevance for patient stratification and immunotherapeutic targeting in ESCC.

### 3.8 Spatial co-expression and compartmentalization of PSD3 and PD-L1 in ESCC tissues

**Figure 8:**
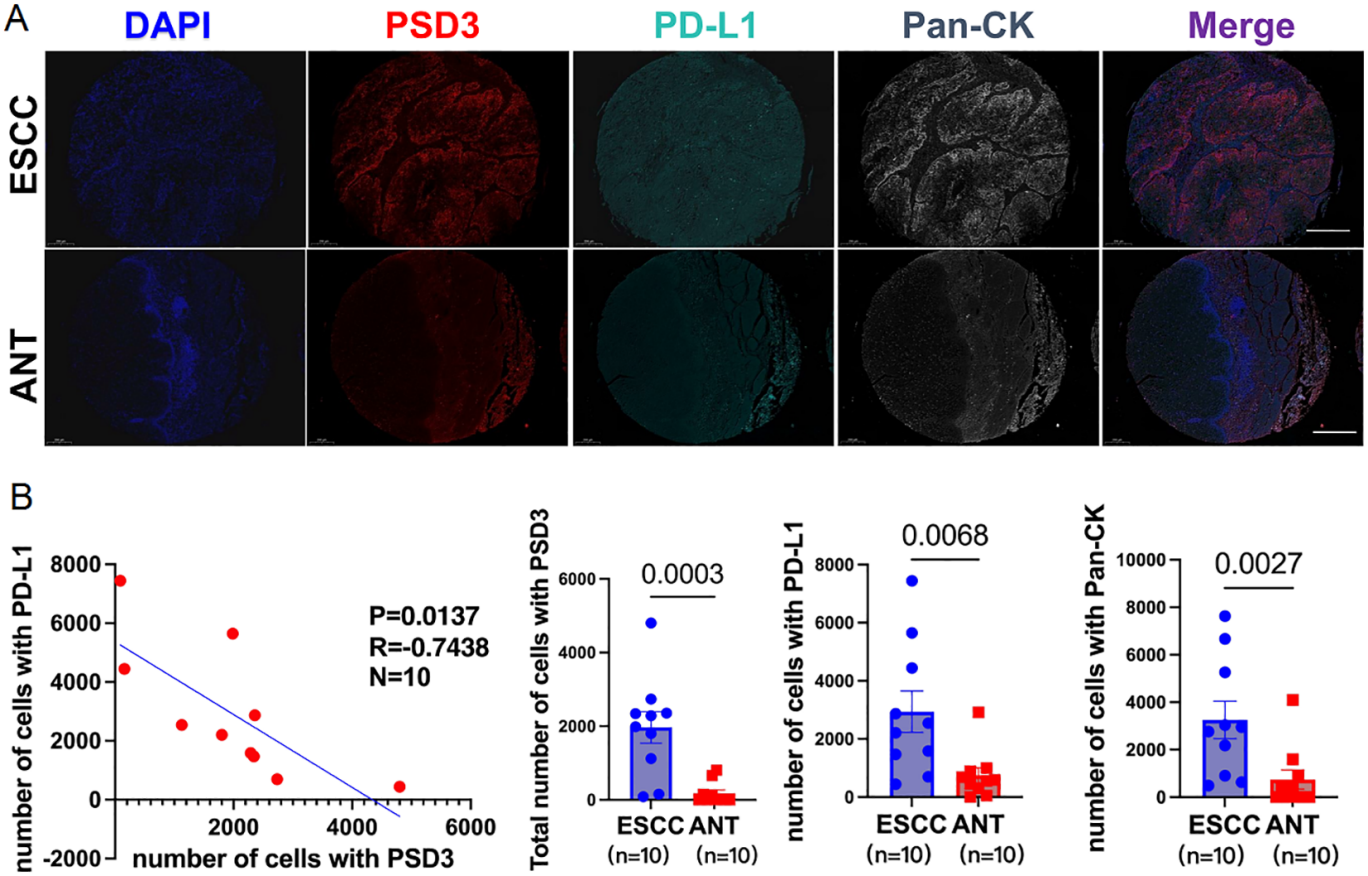
Inverse correlation between PSD3 and CD274 (PD-L1) expression in ESCC, demonstrated by multiplex immunohistochemistry. **(A)** Representative multiplex immunohistochemistry images showing DAPI, PSD3, PD-L1 (CD274), Pan-CK, and merged staining from the same tissue core on an ESCC tissue microarray. ESCC, esophageal squamous cell carcinoma; ANT, adjacent normal tissue; CK, cytokeratin. **(B)** Correlation analysis between PSD3 and PD-L1 expression levels, along with a quantitative comparison of their expression between ESCC and ANT samples. Statistical significance was determined using a two-tailed independent samples t-test.

Following bioinformatic predictions of an inverse relationship between PSD3 and PD-L1, we sought to validate their spatial expression patterns using multiplex immunohistochemistry (mIHC) on a tissue microarray comprising 10 paired ESCC and adjacent normal samples (**Figure 8A**). With the aid of pan-cytokeratin (CK) staining, we were able to accurately delineate epithelial compartments from surrounding stromal regions, allowing for precise localization of protein expression. Notably, both PSD3 and PD-L1 were markedly up-regulated in ESCC tissues compared to their paired normal counterparts. However, despite their elevated levels, a significant inverse correlation between the two proteins was observed across the cohort (r = –0.42, P < 0.01) (**Figure 8B**). This spatially resolved analysis revealed that while both proteins are enriched in tumor regions, their expression tends to be mutually exclusive within the epithelial compartment. Such compartmentalized, negatively correlated expression patterns may hint at a functional interplay or regulatory divergence between PSD3 and PD-L1 in shaping the tumor immune microenvironment. Together, these mIHC results offer supportive histological evidence for the transcriptional antagonism previously suggested by bioinformatic analysis, and underscore the importance of spatial context in understanding immune regulatory dynamics within ESCC tissues.

### 3.9 PSD3 knockdown inhibits malignant phenotypes and up-regulates PD-L1 in murine ESCC cells

**Figure 9:**
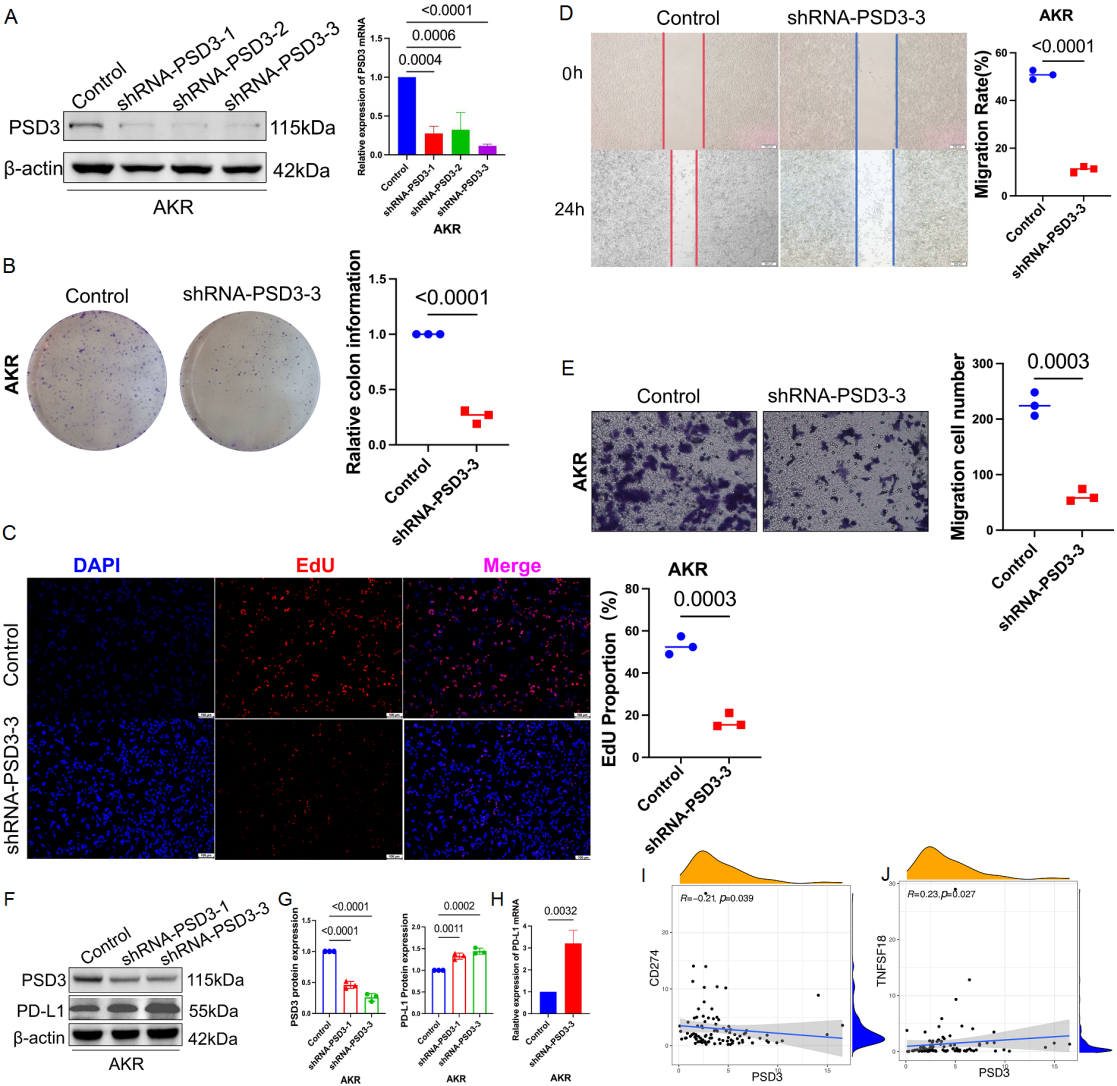
PSD3 promotes malignant behaviors in murine ESCC cells. **(A)** Knockdown efficiency of PSD3 at three distinct target sites in the murine ESCC cell line AKR, demonstrated by Western blot. Statistical analysis was performed using a two-tailed independent samples t-test. **(B)** Silencing of PSD3 significantly suppresses AKR cell proliferation, as shown by clonogenic assay. **(C)** EdU incorporation assay further confirms the inhibitory effect of PSD3 silencing on AKR cell proliferation. **(D)** Wound-healing assay demonstrates that PSD3 knockdown impairs the migratory capacity of AKR cells. **(E)** Transwell invasion assay reveals that PSD3 knockdown reduces the invasive ability of AKR cells compared to control. Statistical significance was assessed using a two-tailed independent samples t-test. **(F)** Knockdown of PSD3 upregulates PD-L1 expression, as demonstrated by immunoblot analysis. **(Gv** Quantitative analysis of band intensities for PD-L1 and PSD3 from the immunoblot shown in panel F. **(H)** Silencing of PSD3 significantly increases PD-L1 mRNA expression, as shown by quantitative real-time PCR (qRT-PCR). **(I, J)** Pearson correlation analyses between PSD3 and PD-L1 expression, and between PSD3 and TNFSF18 expression, in ESCC based on TCGA data.

To assess functional relevance, we knocked down PSD3 in AKR murine ESCC cells using shRNA constructs targeting three loci. Western blot confirmed significant reduction of PSD3 expression (**Figure 9A**). Functionally, PSD3 depletion led to substantial decreases in colony formation (~45%), EdU incorporation (~38%), migration (~42%), and invasion (~50%) (**Figures 9B–E**), demonstrating its role in tumor aggressiveness. Strikingly, PD-L1 expression was markedly up-regulated following PSD3 knockdown, as confirmed by both Western blot and qRT-PCR (**Figures 9F–H**). TCGA data further corroborated this inverse relationship (r = -0.37, P < 0.01), and a parallel positive correlation was noted between PSD3 and TNFSF18 (r = 0.35, P < 0.05; **Figures 9I, J**). These results support a model wherein PSD3 suppresses PD-L1 expression while promoting malignant phenotypes.

### 3.10 PD-L1 knockout restricts tumor cell growth and invasion in human ESCC cells

**Figure 10:**
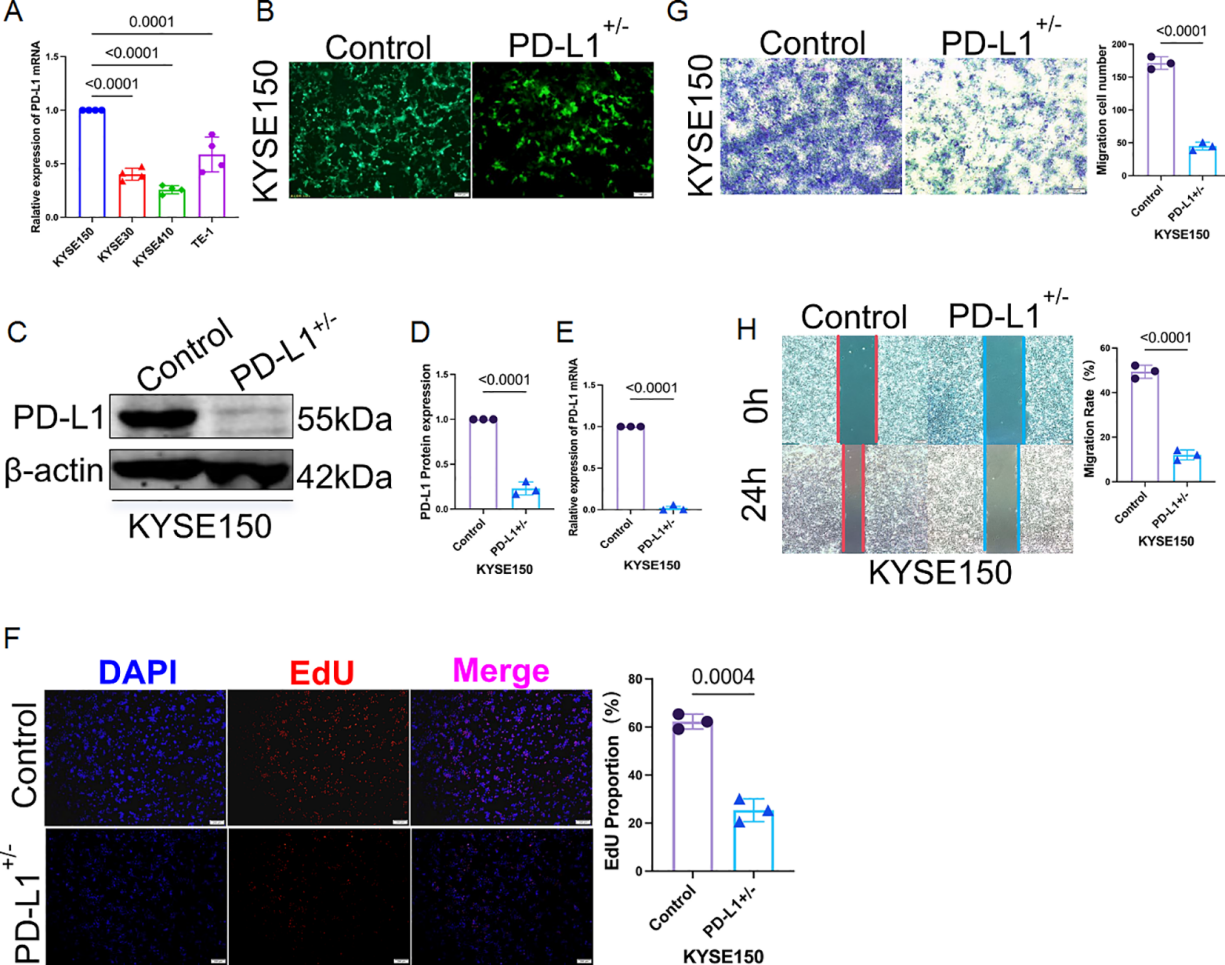
Heterozygous knockout of PD-L1 markedly suppresses proliferation, invasion, and migration of KYSE150 cells. **(A)** Among four human ESCC cell lines (KYSE150, KYSE30, KYSE410, and TE-1), qRT-PCR analysis revealed that KYSE150 exhibited the highest basal mRNA expression level of PD-L1. **(B)** Heterozygous knockout of PD-L1 in KYSE150 cells was established using CRISPR-Cas9 lentiviral transduction. Green fluorescent protein (GFP) indicates successful transfection, with puromycin selection performed over two weeks. **(C)** The knockout efficiency at the protein level was validated by immunoblotting. **(D)** Quantification of PD-L1 band intensity from the immunoblot shown in panel **(C, E)** PD-L1 mRNA expression was also assessed post-knockout using qRT-PCR, confirming reduced transcript levels. **(F)** EdU proliferation assay demonstrated significantly reduced cell proliferation in PD-L1–deficient KYSE150 cells compared to controls. **(G)** Transwell invasion assay showed that PD-L1 heterozygous knockout substantially impaired the invasive capacity of KYSE150 cells. **(H)** Wound-healing assay further confirmed that the migratory ability of PD-L1–knockout KYSE150 cells was markedly reduced.

To validate the functional role of PD-L1, we generated a heterozygous PD-L1 knockout in KYSE150 cells using CRISPR-Cas9, selected due to its high baseline PD-L1 expression (**Figure 10A**). GFP positivity and puromycin selection ensured efficient transfection (**Figure 10B**), with immunoblot and qRT-PCR confirming reduced PD-L1 expression (**Figures 10C–E**). Knockout cells exhibited markedly impaired proliferative capacity (EdU reduction ~40%), as well as diminished invasive and migratory behavior in transwell and wound-healing assays (reductions of ~50% and ~46%, respectively; **Figures 10F–H**). These findings underscore PD-L1’s tumor-promoting role, potentially linked to PSD3-mediated pathways.

### 3.11 PSD3 physically interacts with PD-L1 across species

**Figure 11:**
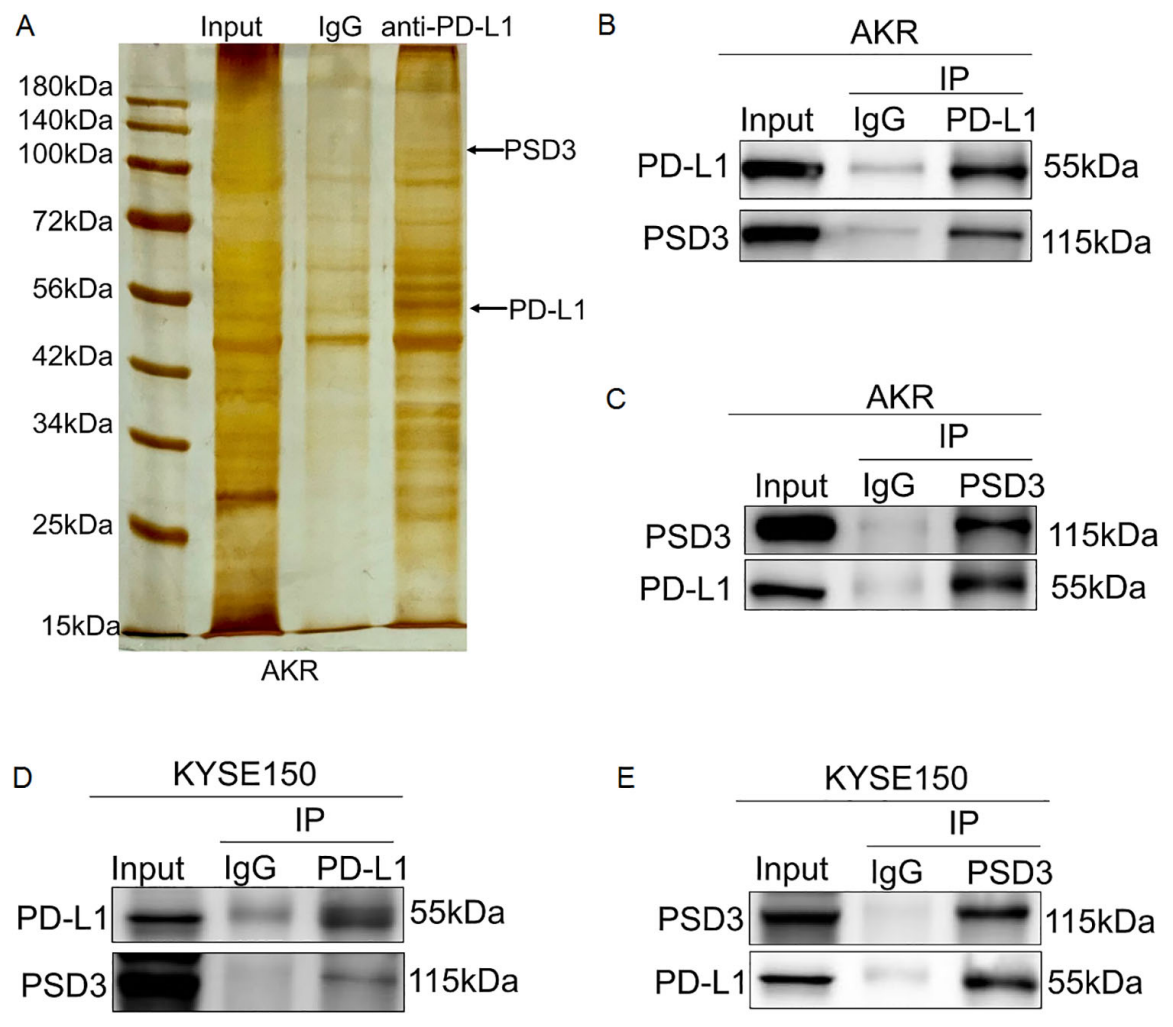
PSD3 physically and directly interacts with PD-L1 in AKR and KYSE150 cells. **(A)** Silver staining of a 10% SDS-PAGE gel showing input, IgG control, and eluates obtained from AKR cells using magnetic beads conjugated with an anti–PD-L1 antibody. **(B, C)** Endogenous co-immunoprecipitation (Co-IP) assays confirmed a direct physical interaction between PD-L1 and PSD3 in AKR cells. **(D, E)** Similar Co-IP experiments in KYSE150 cells demonstrated that PSD3 also directly binds to PD-L1 in human ESCC cells.

Given the inverse regulatory relationship observed, we hypothesized a direct physical interaction between PSD3 and PD-L1. Silver staining and co-immunoprecipitation in AKR cells confirmed that PSD3 could be pulled down using anti-PD-L1 antibodies (**Figures 11A–C**). This interaction was further validated in KYSE150 cells, indicating that the PSD3–PD-L1 binding interface is conserved in both murine and human ESCC systems (**Figures 11D, E**). This direct interaction provides a compelling molecular mechanism linking PSD3 expression to PD-L1 regulation and immune evasion.

## 4 Discussion

In this study, we conducted a comprehensive bioinformatic analysis of three immune-related genes, PSD3, CD274 (PD-L1), and TNFSF18 (GITRL), with the aim of elucidating their potential roles in ESCC. Our findings demonstrate that these genes are significantly up-regulated in ESCC tissues, are associated with poor patient prognosis, correlate with key clinicopathological parameters, and are implicated in immune-related biological pathways and immune cell infiltration. Together, these results position PSD3, CD274, and TNFSF18 as potential diagnostic, prognostic, and immunological biomarkers in ESCC.

The first major finding of our study is the consistently elevated expression of CD274 and TNFSF18 in ESCC tumor tissues compared to normal esophageal epithelium, as observed in both the TCGA and GEO datasets. In contrast, PSD3 was significantly up-regulated in ESCC samples within the TCGA dataset but did not show a significant difference in the GEO dataset, where its expression was slightly lower in tumors than in normal tissues. These findings suggest that while CD274 and TNFSF18 may serve as robust biomarkers across cohorts, the expression pattern of PSD3 may be context-dependent or cohort-specific, warranting further validation in larger, independent datasets. CD274 is well-established as an immune checkpoint molecule that enables tumor immune evasion by binding to PD-1 on T cells (26, 27), thus suppressing their cytotoxic function. TNFSF18, a ligand in the TNF superfamily, is known to modulate T cell activation and proliferation (28, 29). PSD3, though less well-characterized in the context of cancer (10, 30), has been associated with intracellular signaling and may contribute to oncogenic processes through modulation of the cytoskeleton (14) or vesicle trafficking (31).

Secondly, survival analysis revealed that high expression of each of these genes is significantly associated with worse overall survival in ESCC patients. These results indicate that PSD3, CD274, and TNFSF18 are not only over-expressed but also clinically relevant in terms of predicting disease outcome. Furthermore, their expression correlates with advanced T and N stages and higher clinical staging, suggesting a role in tumor aggressiveness. These correlations underline their potential as prognostic biomarkers that could aid in risk stratification and clinical decision-making.

The apparent paradox—where higher PSD3 expression is linked to improved survival despite its tumor-promoting functions *in vitro*—may reflect the gene’s context-dependent roles. One possibility is that PSD3 participates in feedback regulation that indirectly enhances antitumor immune surveillance, possibly by down-regulating PD-L1. Alternatively, tumors with higher PSD3 levels may be more immunologically “visible,” triggering a stronger host immune response despite their aggressive phenotype. Another consideration is that PSD3’s association with favorable prognosis may be confounded by subtype-specific tumor biology or treatment sensitivity. Further investigation into PSD3’s spatial localization, immune contexture, and pathway interactions is warranted.

In addition to prognostic relevance, our ROC curve analyses showed that PSD3, CD274, and TNFSF18 exhibit moderate to high diagnostic accuracy in distinguishing tumor tissues from normal controls. Such performance suggests their potential utility in the early detection or screening of ESCC, particularly in high-risk populations.

To explore the biological underpinnings of these findings, we conducted gene co-expression and enrichment analyses. Genes positively associated with PSD3, CD274, and TNFSF18 were significantly enriched in immune-related processes, including leukocyte activation, cell–cell adhesion, and cytokine signaling. These findings reinforce the concept that the tumor microenvironment in ESCC is strongly immunologically active, and that these genes may play regulatory roles within this context.

Of particular interest is the consistent and robust association between high gene expression and increased infiltration of immune cells, especially T cells and dendritic cells. This observation is particularly relevant for CD274 and TNFSF18, both of which have well-documented immunomodulatory functions (32). The link between high expression and immune infiltration may reflect an adaptive immune response to the tumor or, alternatively, a tumor-driven strategy to co-opt immune signaling for immune evasion. These data echo findings from other malignancies in which PD-L1 and TNFSF18 are implicated in both immune suppression and immune activation depending on the tumor context.

Further dissection of immune cell-type correlations provided additional insight into how PSD3, CD274, and TNFSF18 may shape the tumor immune microenvironment (TME) in ESCC. PSD3 expression was positively associated with activated dendritic cells, resting CD4^+^ memory T cells, and M1 macrophages, cell types typically linked to antigen presentation and pro-inflammatory responses. This immune profile suggests that PSD3 may participate in modulating immune priming and macrophage polarization within the TME, possibly influencing the balance between immune surveillance and immune escape. In contrast, CD274 expression was associated not only with M1 macrophages but also with resting mast cells and regulatory T cells (Tregs), the latter being well-known mediators of immunosuppression. This aligns with the canonical role of PD-L1 in dampening cytotoxic T cell responses and maintaining an immunosuppressive milieu. TNFSF18 showed positive correlations with resting dendritic cells, B cells, and plasma cells, indicating its involvement in humoral immune responses and suggesting a role in B cell activation and differentiation. Collectively, these findings point to distinct yet overlapping immunological footprints for each gene, reinforcing the hypothesis that PSD3, CD274, and TNFSF18 collectively contribute to the dynamic immunoarchitecture of ESCC. Their associations with both stimulatory and suppressive immune populations highlight their dual potential as biomarkers of immune contexture and as functional modulators within the TME.

Interestingly, despite their central roles in anti-tumor immunity, our *in silico* analyses did not reveal significant correlations between the expression of PSD3, CD274, or TNFSF18 and the infiltration of CD8+T cells or natural killer (NK) cells. This finding may reflect methodological constraints inherent to immune deconvolution tools, which rely on bulk transcriptomic data and algorithm-specific gene signatures to infer cell proportions. Variability in data preprocessing, tissue heterogeneity, and limited resolution of certain immune subtypes could contribute to underestimating associations, particularly for cell types like CD8+T and NK cells that often exhibit activation-dependent transcriptional shifts. Moreover, the absence of correlation *in silico* does not preclude functional interactions *in situ*, especially considering the known roles of CD274 and TNFSF18 in modulating cytotoxic responses. Thus, it is plausible that PSD3, CD274, and TNFSF18 influence the activity or functional phenotype of CD8+T and NK cells rather than their infiltration level *per se*. This warrants further investigation using orthogonal experimental approaches such as mIHC, spatial transcriptomics, or single-cell RNA sequencing to better resolve the spatial and functional interplay between these immune-regulatory genes and cytotoxic lymphocyte populations within the ESCC microenvironment.

Building upon the computational analyses and immune correlation findings, our mIHC validation in a small cohort of 10 paired ESCC and adjacent normal tissue samples revealed a striking inverse correlation between PSD3 and PD-L1 (CD274) expression. The spatially distinct and negatively correlated expression patterns suggest that PSD3 may exert an inhibitory effect on PD-L1, potentially modulating the tumor’s capacity for immune evasion. While PD-L1 is well established as an immunosuppressive ligand that interacts with PD-1 on T cells to dampen anti-tumor immunity, the observed inverse association implies that high PSD3 expression may coincide with a less immunoevasive phenotype, or alternatively, reflect a compensatory regulatory mechanism within the tumor microenvironment. Although the small sample size limits definitive conclusions, this finding offers a compelling direction for future mechanistic studies exploring PSD3’s role in immune checkpoint regulation.

In addition to spatial correlation data, our *in vitro* experiments using murine ESCC cells provide the first functional evidence that PSD3 plays a causative role in driving tumor aggressiveness. Knockdown of PSD3 significantly impaired proliferation, migration, and invasion of AKR cells, indicating that PSD3 facilitates key malignant behaviors in ESCC. To further validate our findings in a human ESCC context, we introduced the KYSE150 cell line, which exhibited the highest basal PD-L1 expression among the four tested human ESCC lines. This enabled us to functionally explore PD-L1’s role through gene editing while maintaining translational relevance.

In our functional assays, we selected the murine ESCC cell line AKR, along with a human-derived cell line KYSE150. This choice was guided by strategic translational considerations: using a syngeneic murine system enables downstream *in vivo* studies within immunocompetent mouse models, such as C57BL/6N mice (33, 34). Given that PSD3 appears to have immunological relevance, particularly in shaping immune evasion and tumor-immune interactions, the use of a murine model provides a crucial advantage for future mechanistic studies. It allows for the dynamic assessment of PSD3’s role in tumor growth, immune cell infiltration, and potential response to immunotherapy in a physiologically relevant, immune-intact host environment. Thus, the AKR cell model lays the foundation for extending our findings from *in vitro* observations to *in vivo* immunological validation.

These results are consistent with one prior study (10) implicating PSD3 acted as oncogene in papillary thyroid cancer, which promoted malignant behaviors of papillary thyroid cancer cells. Importantly, these findings also align with our earlier enrichment analyses suggesting PSD3’s involvement in immune and inflammatory pathways. Taken together, the evidence positions PSD3 not only as a biomarker with diagnostic and prognostic relevance but also as a putative driver of ESCC progression with immunological significance.

Given these multifaceted roles, PSD3 emerges as a particularly attractive candidate for further investigation in the context of both tumor biology and cancer immunotherapy. To gain mechanistic insight into the relationship between PSD3 and immune modulation, we performed targeted experiments to assess the regulatory interplay between PSD3 and PD-L1. Notably, PSD3 knockdown in murine ESCC cells (AKR) resulted in a marked up-regulation of PD-L1 expression at both the protein and mRNA levels (**Figures 9F–H**). These findings were further supported by a significant inverse correlation between PSD3 and PD-L1 expression in the TCGA ESCC cohort (**Figure 9I**). Interestingly, we also observed a positive correlation between PSD3 and TNFSF18 (**Figure 9J**), suggesting a broader immunoregulatory network involving PSD3.

Functionally, heterozygous knockout of PD-L1 in KYSE150 cells, selected for their high endogenous PD-L1 expression, led to profound reductions in cellular proliferation, migration, and invasion (**Figures 10A–H**). These results not only confirm the pro-tumorigenic role of PD-L1 in ESCC, but also imply that its suppression via PSD3 may be part of a compensatory regulatory mechanism that favors immune evasion through PD-L1-independent routes.

Most strikingly, our co-immunoprecipitation experiments demonstrated that PSD3 physically interacts with PD-L1 in both murine and human ESCC cell lines (**Figures 11A–E**), providing direct biochemical evidence of their association. This protein–protein interaction may represent a previously unrecognized checkpoint regulatory axis, where PSD3 negatively modulates PD-L1 stability or localization. Collectively, these findings support a dual functional role for PSD3 in ESCC: promoting oncogenic behavior while concurrently influencing the immunological phenotype of tumor cells through modulation of PD-L1.

PSD3’s involvement in immune regulation remains less clear; however, its strong correlation with immune cell infiltration and immune pathway enrichment suggests a previously underappreciated role in modulating the tumor immune landscape. Further experimental studies will be required to validate and elucidate the mechanisms of PSD3 action in immune-oncology.

While our results are robust and align with emerging themes in cancer immunobiology, alternative explanations must be considered. For example, increased immune infiltration associated with high gene expression might not be causally mediated by PSD3, CD274, or TNFSF18, but rather reflect a broader inflammatory state of the tumor (35). Additionally, the survival disadvantage associated with high gene expression could be confounded by other unmeasured confounding molecular or clinical factors, such as treatment history, molecular subtypes, or comorbidities, which are not captured in the current analysis (36).

Our study also has inherent limitations. First, it relies exclusively on publicly available datasets from TCGA, which, although comprehensive, may include batch effects (37), population biases (38, 39), and incomplete clinical annotations (40). Second, our findings are correlative and lack functional validation through experimental assays. Third, the lack of independent external validation cohorts (41) limits the generalizability of our conclusions. Lastly, while CD274 and TNFSF18 are well-characterized in immune signaling, PSD3’s role in cancer remains poorly defined and should be interpreted with caution.

Despite these limitations, our findings have clear clinical implications. The association of PSD3, CD274, and TNFSF18 with survival, staging, and immune cell infiltration underscores their potential as biomarkers for prognostic assessment and therapeutic targeting. In particular, CD274 is already the basis of FDA-approved immune checkpoint inhibitors in various cancers. Whether PSD3 or TNFSF18 could serve as novel immunotherapy targets, or as predictive biomarkers for immunotherapy response, warrants further investigation.

Future studies should focus on the mechanistic validation of PSD3 in ESCC progression and immunity. *In vivo* experiments and patient-derived tissue analyses are needed to clarify causal relationships. In addition, integrating transcriptomic findings with proteomic, single-cell, and spatial data could provide a more granular understanding of the tumor microenvironment.

Taken together, our findings offer a nuanced view of how PSD3, CD274, and TNFSF18 contribute to the immune landscape and molecular pathology of ESCC. Rather than serving as uniformly prognostic markers or immune targets, these genes demonstrate divergent expression dynamics, immune associations, and clinical implications in ESCC. CD274 and TNFSF18 were consistently up-regulated in tumor tissues across both TCGA and GEO cohorts, whereas PSD3 exhibited a context-dependent pattern, showing significant up-regulation in TCGA but no differential expression in GEO. Among the three, only PSD3 expression was significantly associated with overall survival, with higher levels correlating with favorable prognosis. Immune infiltration analyses revealed that PSD3 was negatively associated with immunosuppressive cell types such as regulatory T cells and M1 macrophages, while CD274 was positively linked to Tregs and resting mast cells. TNFSF18 showed enrichment patterns involving B cells, plasma cells, and resting dendritic cells. These findings reflect the distinct and heterogeneous roles of these genes in shaping the immune landscape of ESCC, with PSD3 emerging as a context-dependent immunomodulatory factor with unexpected protective associations.

Importantly, mIHC confirmed an inverse spatial correlation between PSD3 and CD274 protein expression in tumor tissues, suggesting potential regulatory crosstalk relevant to immune evasion. Functional *in vitro* experiments further demonstrated that silencing PSD3 impairs ESCC cell proliferation, migration, and invasion, indicating a direct oncogenic role. However, contrary to expectations, no significant associations were observed between the expression of these genes and the infiltration of CD8+T or NK cells, highlighting the limitations of computational deconvolution and the need for higher-resolution, spatially contextualized validation.

In summary, our study identifies PSD3 as a novel, context-dependent immuno-oncogenic factor in esophageal squamous cell carcinoma (ESCC). While PSD3, CD274, and TNFSF18 were found to be up-regulated in ESCC and associated with immune-related features, only PSD3 demonstrated a significant and paradoxical correlation with patient survival—higher expression predicting better prognosis. Functional assays revealed that PSD3 promotes tumor cell proliferation, migration, and invasion while negatively regulating PD-L1 expression. Furthermore, we uncovered a direct physical interaction between PSD3 and PD-L1, suggesting a novel regulatory mechanism that may influence immune escape. These findings not only establish PSD3 as a potential biomarker and therapeutic target in ESCC but also provide mechanistic insight into how immune checkpoint dynamics may be modulated by less-characterized oncogenic drivers.

## Data Availability

The datasets presented in this study can be found in online repositories. The names of the repository/repositories and accession number(s) can be found in the article/supplementary material.

## Glossary

CD274: Cluster of Differentiation 274 (also known as PD-L1)
PSD3: Pleckstrin and Sec7 Domain Containing 3
TNFSF18: Tumor Necrosis Factor (Ligand) Superfamily Member 18
OS: Overall Survival
AUC: Area Under the Curve
CI: Confidence Interval
GSEA: Gene Set Enrichment Analysis
NES: Normalized Enrichment Score
FDR: False Discovery Rate
NFE2L2: Nuclear Factor Erythroid 2–Like 2
GO: Gene Ontology
ACC: Adrenocortical Carcinoma
BLCA: Bladder Urothelial Carcinoma
BRCA: Breast Invasive Carcinoma
CESC: Cervical Squamous Cell Carcinoma and Endocervical Adenocarcinoma
CHOL: Cholangiocarcinoma
COAD: Colon Adenocarcinoma
DLBC: Lymphoid Neoplasm Diffuse Large B-cell Lymphoma
ESCC: Esophageal Squamous Cell Carcinoma
GBM: Glioblastoma Multiforme
HNSC: Head and Neck Squamous Cell Carcinoma
KICH: Kidney Chromophobe
KIRC: Kidney Renal Clear Cell Carcinoma
KIRP: Kidney Renal Papillary Cell Carcinoma
LAML: Acute Myeloid Leukemia
LGG: Brain Lower Grade Glioma
LIHC: Liver Hepatocellular Carcinoma
LUAD: Lung Adenocarcinoma
LUSC: Lung Squamous Cell Carcinoma
MESO: Mesothelioma
OV: Ovarian Serous Cystadenocarcinoma
PAAD: Pancreatic Adenocarcinoma
PCPG: Pheochromocytoma and Paraganglioma
PRAD: Prostate Adenocarcinoma
READ: Rectum Adenocarcinoma
SARC: Sarcoma
SKCM: Skin Cutaneous Melanoma
STAD: Stomach Adenocarcinoma
TGCT: Testicular Germ Cell Tumors
THCA: Thyroid Carcinoma
THYM: Thymoma
UCEC: Uterine Corpus Endometrial Carcinoma
UCS: Uterine Carcinosarcoma
UVM: Uveal Melanoma.
